# Further Evidence of Population Admixture in the Serbian Honey Bee Population

**DOI:** 10.3390/insects13020180

**Published:** 2022-02-09

**Authors:** Marija Tanasković, Pavle Erić, Aleksandra Patenković, Katarina Erić, Milica Mihajlović, Vanja Tanasić, Szilvia Kusza, Andrzej Oleksa, Ljubiša Stanisavljević, Slobodan Davidović

**Affiliations:** 1 Department of Genetics of Populations and Ecogenotoxicology, Institute for Biological Research “Siniša Stanković”—National Institute of the Republic of Serbia, University of Belgrade, Bulevar Despota Stefana 142, 11060 Belgrade, Serbia; pavle.eric@ibiss.bg.ac.rs (P.E.); aleksandra@ibiss.bg.ac.rs (A.P.); katarina.eric@ibiss.bg.ac.rs (K.E.); slobodan.davidovic@ibiss.bg.ac.rs (S.D.); 2 Center for Forensic and Applied Molecular Genetics, Faculty of Biology, University of Belgrade, Studentski trg 16, 11000 Belgrade, Serbia; milica.mihajlovic@bio.bg.ac.rs (M.M.); vanja.tanasic@bio.bg.ac.rs (V.T.); 3 Centre for Agricultural Genomics and Biotechnology, University of Debrecen, Egyetem tér 1., 4032 Debrecen, Hungary; kusza@agr.unideb.hu; 4 Department of Genetics, Faculty of Biological Sciences, Kazimierz Wielki University, Powstanców Wielkopolskich 10, 85-090 Bydgoszcz, Poland; olek@ukw.edu.pl; 5 Faculty of Biology, University of Belgrade, Studentski trg 16, 11000 Belgrade, Serbia; ljstanis@bio.bg.ac.rs

**Keywords:** honey bee, microsatellite, population genetics, genetic diversity

## Abstract

**Simple Summary:**

The western honey bee is one of the most ecologically and economically important pollinator species. Due to human interference, it faces serious challenges, not only in number decline and habitat loss, but also in natural subspecies diversity and distribution. The conservation of genetic diversity and perseverance of locally adapted populations and subspecies becomes a crucial task in the face of rapid environmental changes. In order to further assess present genetic variability in Serbian honey bee populations, we analyzed 14 microsatellite loci and then compared nine of them with previously published data. Our results suggest that Serbia now harbors a distinct, relatively homogenous honey bee population, although some local differences are still preserved.

**Abstract:**

Socioeconomic interests and beekeeper preferences have often taken precedence over the conservation of locally native honey bee subspecies, leading to the predominance of admixture populations in human-dominated areas. To assess the genetic diversity of contemporary managed Serbian honey bee colonies, we used 14 microsatellite loci and analyzed 237 worker bees from 46 apiaries in eight localities of northern and southern Serbia. Furthermore, we compared data for nine microsatellite loci with 338 individuals from Italy, Hungary, Poland, and Spain. The standard parameters of genetic diversity in Serbian honey bee populations were in line with other analyses, although somewhat smaller. STRUCTURE analysis showed the existence of two equally distributed genetic clusters and Analysis of molecular variances could not confirm the presence of a geographically discrete population but showed local differences. Discriminant analysis of principal components showed overlapping of worker bees from different parts of Serbia. Clear genetic differentiation can be observed when comparing all populations between geographical regions and their corresponding subspecies. The absence of the *A. m. macedonica* subspecies from its historical distribution range in southern Serbia as well as the lack of distinctive geographical groups suggest that selective breeding, queen import, and migratory beekeeping practices strongly influence the genetic structure and diversity of honey bees, leading to the genetic uniformization and creation of the admixture population.

## 1. Introduction

The western honey bee (*Apis mellifera* Linnaeus, 1758) is one of the species that is a subject of constant human interference. Although its domestication began more than 10,000 years ago, this species has never become truly domesticated despite all efforts, mainly due to its complex mating behavior [1,2,3]. The species’ native range of distribution in western Asia, Africa, and Europe was expanded to all other continents, except Antarctica, to regions marked by highly distinct ecological and climate conditions. In addition to large-scale, transcontinental movements and expansion of the natural range, beekeeping practices and preferences for perceptively more suitable subspecies significantly changed distribution and variability in the historical range of distribution. In recent years, a substantial body of evidence has confirmed that, deliberately or not, humans shape the current diversity of honey bees worldwide [4].

Since the classical Ruttner categorization of the *A. mellifera* subspecies [5], there has been an ongoing debate about its taxonomy, number of subspecies, distribution range, and origin due to the specificity of population structure, features of biology, and resolutions of honey bee subspecies discrimination methods. Based on the results of genomic analysis [6,7,8,9], it was proposed that this species originated in northern Africa or the Middle East [10,11], but the most recent work [12] showed adaptive radiation of subspecies from Asia. From there, it colonized its native geographic range, and followed by multiple colonization waves and glacial events, it diverged into 33 existing subspecies [13] and divided into five evolutionary lineages (A, C, M, O, and Y). However, it still remains unclear how accurate this number of subspecies is, since many subspecies, due to high phenotypic plasticity, have ecotypes previously defined as subspecies [4,14,15,16]. The main differences between subspecies, often referred to as geographic races, are most likely the result of both local adaptations to distinct environments and geographical isolation. However, reproductive isolation does not often exist, and subspecies readily interbreed when they come into contact, although partial reproductive isolation is observed [17]. When subspecies come into contact, naturally or by human interference, it is inevitable that an admixture population will be established and that introgression of foreign genetic variants can be detected in native populations. This situation is especially prominent in Europe, which is the natural area of distribution of mitochondrial lineages A, M, and C. Beekeepers prefer subspecies *A. m. carnica,* and *A. m. ligustica*, both classified as C lineage, and their Buckfast hybrid has been imported to almost all parts of the continent [18,19,20,21]. It resulted in a considerable degree of genetic admixture among subspecies, even though in some areas the specific genetic footprint of autochthonous subspecies is still preserved [22,23,24,25,26]. For example, *A. m. mellifera* populations (belonging to the M lineage) have been hybridized in varying degrees in most of their native areas and in some parts, such as Germany, have been almost replaced by *A. m. carnica* because local beekeepers preferred this subspecies [27]. The loss of native subspecies and specific genetic diversity they harbor made conservationists and lawmakers in several countries establish protected areas for their native subspecies (Denmark and Great Britain) [19] as well as make laws that prohibit the breeding of nonnative bees (for example, Serbia and Croatia) [28].

Due to the spread of Varroa mites in the last decades of the twentieth century, it was believed that wild honey bee colonies became extinct in Europe, but now new evidence is emerging that some areas have thriving feral or even possibly wild honey bee colonies [29,30,31,32]. Therefore, it is not surprising that most research on the genetics of honey bees is conducted in managed colonies. Large-scale genetic comparisons show that genetic structure in any given area is heavily dependent on several factors, the most prominent of all being the level of importation of foreign queens and the presence and the type of breeding strategy implemented by beekeepers and their organizations. Extensive management by beekeepers promotes population admixture [33], which is expected when humans facilitate the movement and interbreeding of previously structured populations [34]. The general conclusion is that many lines used for contemporary beekeeping in Europe consist of a mixture of different source populations [35]. The bees from areas with frequent queen importation show a high level of admixture and are hardly assignable to distinct subspecies anymore, but for those in areas where breeding lines were selected and maintained at their geographical origin, genetic identity was preserved and they resemble their native source populations [28,31,36,37,38,39].

It is noted that in areas that are natural contact zones of different *A. mellifera* subspecies, natural hybridization occurs [40,41] and the hybridization is inevitable in regions where human interference due to beekeeper preferences is high, which occurred in the C lineage native area of distribution [24,25,26,42,43]. Serbia, located in the center of the Balkan Peninsula, is geographically in the middle of the distribution range of the C lineage. Previous analysis showed that of four C lineage subspecies, Serbia harbors two (*A. m. macedonica* and *A. m. carnica*) which are clinaly distributed from the northwest (*carnica*) to the southeast (*macedonica*) with a hybrid zone between them [23,26,44,45]. Furthermore, nine described mtDNA haplotypes for *tRNA^leu^-cox2* of which two are novel [46] and three distinct ecotypes belonging to specific geographical regions [47] reflect significant genetic variability of *A. mellifera* in this region. In the past 30 years, the variability of Serbian honey bees has been extensively described on morphological [26,48], etiological [49,50], and genetic levels [26,44,45,47,51,52,53]. Microsatellite analysis of Serbian honey bees from the first decade of the 21st century showed that although substantial admixture between *A. m. carnica* and *A. m. macedonica* in the central part of Serbia can be detected, populations from the northwest and southeast retain a distinctive subspecies genetic footprint [26]. The results of microsatellite and tRNA^leu^-cox2 mtDNA variability [23] confirmed this clinal distribution of subspecies and their hybrids, with worker bees from northern Serbia forming a distinct genetic cluster characterized as *carnica-2* ecotype, and those from southern Serbia forming a different distinct genetic cluster characterized as the *macedonica*-1 ecotype.

However, beekeeping practices in Serbia have changed dramatically in the last decade. First, the number of managed beehives has doubled since 2009 [54], partly due to the government’s financial support, and now Serbia has the largest number of beehives per capita worldwide (one beehive per six inhabitants). According to our field data, the traditional way of beekeeping is lost and the number of stationary apiaries is dwindling. The production of beekeepers who prefer *A. m. carnica* queens intensified, and the number of queen breeding institutions focused on its desired traits is growing. Serbia is one of the countries that has embedded in its legislation the preservation of autochthonous species, subspecies, and races, and as per the Law on Animal Breeding from 2009, breeding and keeping of only *A. m. carnica* are allowed in its territory [55]. Recent work based on the variability of the tRNA^leu^-cox2 mtDNA region [46] suggests that the composition and distribution of honey bee populations in Serbia has changed over the past decade, invoking the need for further examination of genetic variability on various levels.

Biparental inherited microsatellite loci proved to be an excellent genetic marker for inferring overall population genetic variability, deciphering the distribution of different *A. mellifera* subspecies [19,56], detecting population admixture [7,26], and determining the presence of distinct locally adapted populations [20,57]. Large-scale analysis of microsatellite loci enables a better understanding of large and fine-scale population differentiation. To better understand the genetic variability of contemporary Serbian honey bee populations, we analyzed 14 microsatellite loci in 237 worker bees from the northern and southern parts of Serbia. In addition, we compared our data for nine microsatellite loci with previously published data [58] for 338 individuals from Hungary, Poland, Spain, and Italy belonging to *A. m. mellifera, A. m. carnica*, *A. m. iberiensis*, *A. m. ligustica*, and the Buckfast hybrid.

## 2. Materials and Methods

### 2.1. Sampling

**Figure 1:**
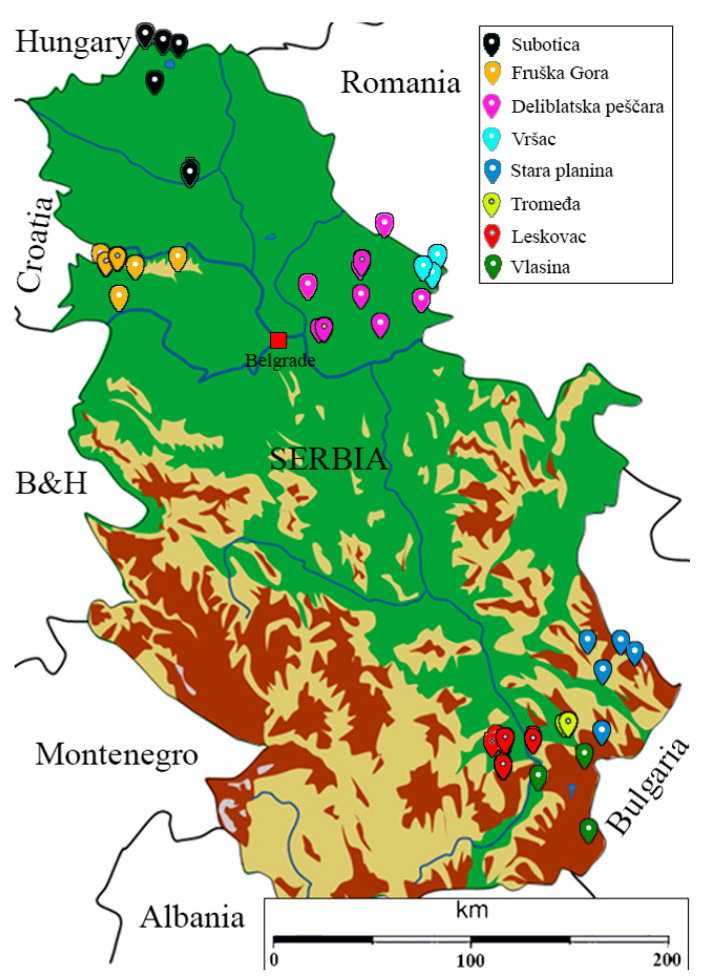
Sampling localities (from [46]).

A total of 237 worker bees were collected from 46 stationary apiaries during late August and early September in 2020 and stored in 95% ethanol at −20 °C for further analysis. Eight localities from southern and northern parts of Serbia were chosen, four in the south (Leskovac (L), Vlasina (V), Stara Planina (SP), and Tromeđa (T)) and four in the north (Subotica (S), Vršac (Vr), Deliblatska pesščcara (DS), and Fruška Gora (FG) (Figure 1). Approximately five worker bees from the apiary were chosen for genetic analysis, each representing one beehive. The detailed specification of sampling sites can be found in [46]. Furthermore, the DNA of 338 individuals from Hungary, Poland, Spain, and Italy belonging to *A. m. mellifera, A. m. carnica*, *A. m. iberiensis*, *A. m. ligustica,* and Buckfast hybrid from Péntek-Zakar 2015 were obtained for comparison.

### 2.2. DNA Extraction and PCR-RFLP Analysis

Whole-genomic DNA was extracted using the protocol described in [59]. The concentration of the extracted DNA and its quality were checked both with a spectrophotometer (NanoPhotometer, IMPLEN, Germany) and an agarose gelelectrophoresis.

The PCR-RFLP method described by [42] was used to distinguish *A. m. carnica* from *A. m. macedonica*. For the amplification of mtDNA COI fragment, the following primers were used: 5′-GATTACTTCCTCCCTCATTA-3′ [60] and 5′-AATCTGGATAGTCTGAATAA-3′ [53]. The PCR amplification of the COI fragment and subsequent digestion with *Nco*I and *Sty*I restriction enzymes were performed according to the protocol described in [46,61].

### 2.3. Microsatellite Analysis

For comparison of different honey bee colonies on the autosomal level, we have 14 microsatellite loci described in [61]. The choice of loci used in the analysis was made according to the most frequent microsatellite loci used in a number of different studies dealing with the variability of these genetic markers. The selected loci, primer pairs used for amplification, and the corresponding annealing temperatures are presented in Table S1. The microsatellite loci were amplified in PCR reactions in which forward primers were labeled with a fluorescent dye (Table S1). PCR was performed in four reactions that differed in annealing temperature (Table S1) using the following program: one cycle of initial denaturation at 94 °C for 5 min, after which there were 30 cycles of 35 s at 94 °C, 35 s at annealing temperature (Table S1) and 35 s at 72 °C. The final elongation step was performed at 72 °C for one hour. Loci were amplified in a MiniAmp Plus Thermal Cycler (Applied Biosystems, ThermoFisher Scientific) in four multiplex reactions. The amplification was carried out in a volume of 20 μL with the following final concentrations of reaction components: 1 × Taq Buffer with (NH_4_)_2_SO_4_, 2.5 mM MgCl_2_, 0.8 mM dNTP mix, 1 U of reverse Taq polymerase (all components were produced by Thermo Fisher Scientific, EU), and 5 pmol of each forward and reverse primer. For the amplification of the microsatellite loci, 1.5–1.9 ng of DNA was used. To verify the reliability of the data, 10% of samples were reamplified for the second time.

To use data from the study [58] we performed calibration by reanalyzing 10 samples and 9 microsatellite loci from this data set. The DNA of the same worker honey bees used in [58] was processed in the same way as the samples from Serbia.

### 2.4. Fragment Analysis

For fragment analysis, the first and second multiplex reactions were multipooled, and the third and fourth multiplex reactions were multipooled. All reactions were mixed in equal volumes and plated as one reaction in a volume of 1 μL. Each amplification mix contained seven different loci. GeneScan 600 LIZ size standard was used to score alleles (Applied Biosystems, Warrington, UK). Fragment analysis was performed on the 3130 Genetic Analyzer (Applied Biosystems, UK). Data were analyzed using Gene Mapper Software (Life Technologies, Foster City, CA, USA).

### 2.5. Statistical Analyses

The standard parameters of genetic diversity for microsatellite loci (number of alleles, allelic size range, average gene diversity over loci, number of alleles based on a minimal sample size (obtained by rarefaction), number of private alleles based on a minimal sample size (obtained by rarefaction), observed (H_O_) and expected (H_E_) heterozygosity, random match probability (RMP), and the mean number of pairwise differences (MPD)) were calculated using Arlequin ver. 3.5.2.2 software [62] and HP-Rare 1.1 [63]. The RMP parameter is used to express the probability that two randomly sampled individuals from a population have a matching genotype and is calculated as the sum of square frequencies [64]. MPD is a parameter that represents the measure of differences between all pairs of haplotypes in the sample. Arlequin ver. 3.5.2.2 software was also used to assess genetic differentiation among populations by analysis of molecular variances (AMOVA) and to estimate the pairwise population and overall *F_ST_* and *F_IS_* values. The statistical significance of all performed tests was assessed with 10,000 permutations. The matrix of pairwise population *F_ST_* values was visualized using a multidimensional scaling method (nonmetric MDS) implemented in the PAST 3.25 software [65], and the R functions connected with Arlequin ver. 3.5.2.2 software. The Hardy–Weinberg equilibrium was tested using Arlequin ver. 3.5.2.2 software with 1,000,000 steps in MC and 100,000 dememorization steps. To correct the probabilities when multiple tests were performed simultaneously, we performed a sequential Bonferroni test for the Hardy–Weinberg equilibrium. The linkage disequilibrium between the pairs of loci was estimated using the likelihood ratio test in Arlequin ver. 3.5.2.2 software with 10,000 steps in MC and 10,000 dememorization steps.

The number of genetic clusters represented in the sample was estimated with STRUCTURE v 2.3.4 software [66,67,68]. For the analysis, the admixture model was used with a burn length of 10,000 and a Markov chain Monte Carlo (MCMC) of 100,000 randomizations. The range of the possible number of clusters (K) was from 1 to 10, with a series of 10 runs for each K. The results obtained by STRUCTURE were analyzed by the STRUCTURE harvester [69]. To detect the number of K groups that best fit the data set, this software used results generated by the STRUCTURE software to create a plot of the mean likelihood value per K value and calculated the highest value of the second-order rate of change (Δ K) using the of Evanno method [70]. The model choice criterion, LnP (D), implemented in the STRUCTURE which detects the true K as an estimate of the posterior probability of the data for a given K, was evaluated as well. The most likely scenario was chosen and used to graphically plot both the individuals and populations analyzed.

The observed distances among samples are presented using discriminant analysis of principal components (DAPC) [71]. This method consists of performing the linear discriminant analysis (LDA) on the principal components analysis’ (PCA) transformed matrix. In the case of samples from Serbia, only LDA was performed on the first 32 PCs and in the case of all analyzed populations on the first 55 PCs which cumulatively conserve 98.9% of the total variance. The number of retained PCs was estimated using randomly repeated cross-validation (100 iterations), which consisted of performing DAPC on 90% randomly sampled training set observations (stratified sampling was used so the training set consisted of 90% of the observations from each population) after retaining 10–183 PCs and using the obtained model to predict the groups (populations) in the remaining 10% of samples (test set). Average prediction success per group was used as a metric. Additionally, the PCA transformed matrix (all 183 PCs) was used to find the optimal number of clusters using Ward’s method [72]. We tested 2–50 clusters, and the optimal number of clusters was chosen using BIC statistics using the “diffNgroup” method. This method uses Ward’s clustering method to split the differences between successive values of the BIC summary statistic into two groups to differentiate sharp decreases from mild decreases or increases. The retained K was the one before the first group switch. Thus, estimated clusters of samples were compared with the a priori defined populations.

## 3. Results

### 3.1. PCR-RFLP

The size of the PCR-amplified COI segment for the RFLP analysis was 1029 bp. Digestion with both *Nco*I and *Sty*I did not show a restriction pattern characteristic for mtDNA lineage found in *A. m. macedonica*. Since no restriction sites were observed after RFLP analysis, we presume that all individuals in our sample belong to *A. m. carnica* [46,61].

### 3.2. Genetic Diversity Analysis

#### 3.2.1. Genetic Diversity Analysis for 14 Microsatellite Loci in the Serbian Sample

The standard diversity parameters for the sampled localities in Serbia for all 14 analyzed microsatellite loci are presented in Table 1 and Table S2. The average numbers of alleles’ observed heterozygosity and average gene diversity over loci were the highest in L, the lowest values for these parameters were found in T, Vr, and FG, respectively. Considering the mean number of private alleles based on minimal sample size, the highest number was observed in L, but values in other analyzed localities were in the same range. The observed heterozygosity was generally lower than expected and *F_IS_* values varied between −0.04 in T and 0.19 in Vr (Table S3). It is interesting to note that the departure from the Hardy–Weinberg equilibrium coincided with significant heterozygote deficiencies, especially for locus A43 in all localities except FG. Furthermore, in all localities except L, observed heterozygosity before Bonferroni corrections for selectively adaptive locus Ap249 was significantly lower than expected (Table S2), and after correction it remained significant for T, FG, S, and DP. Linkage disequilibrium was also observed for some pairs of loci, mostly prominent for two selectively adaptive loci (Ap249 and B124) in three northern and one southern locality (Table S4).

#### 3.2.2. Population Genetic Analysis for Nine Microsatellite Loci in All Sampled Localities

The standard diversity parameters for all sampled localities for nine analyzed microsatellite loci are presented in Table 2 and Table S5. The average number of alleles was the highest in Hungary and the lowest in the Polish sampled site, Wroclaw. The average gene diversity over loci was the highest in Poland and Spain and the lowest in Serbian populations. Heterozygosity excess was observed in all populations except those in Serbia, both for all analyzed loci and individual loci per population as well as for *F_IS_* values (Tables S5 and S6). Linkage disequilibrium analysis was also performed for this set of data and the results are presented in Table S7.

### 3.3. Population Structure

#### 3.3.1. Population Structure Based on 14 Microsatellite Loci in the Serbian Sample

**Figure 2:**
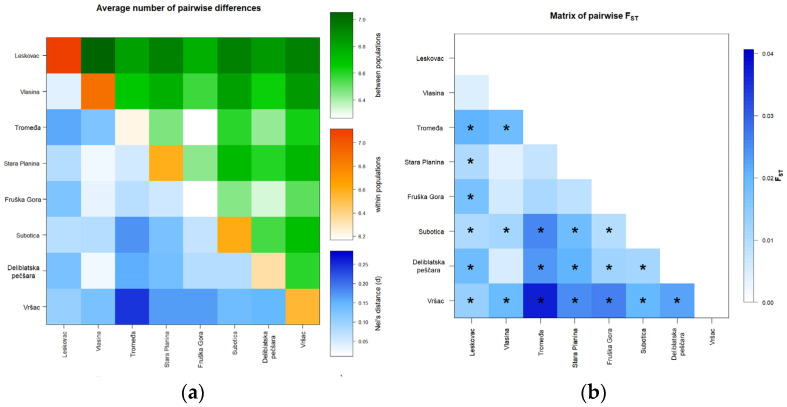
Matrixes of the average number of pairwise *Nei’s* (**a**) and *F_ST_* (**b**) distances based on the analysis of 14 microsatellite loci for localities in Serbia. (**a**) The average number of pairwise differences between populations is presented above diagonally, the average number of pairwise differences within the population is presented diagonally, and *Nei’s* distances are presented below diagonally. (**b**) Statistically significant *F_ST_* values are marked with an asterisk (*). Part of the results are published in Proceedings of the The 1st International Electronic Conference on Entomology session Apiculture and Pollinators, 1–15 July 2021, MDPI: Basel, Switzerland, doi: 10.3390/IECE-10720.

The average number of pairwise differences between and within Serbian localities together with *Nei’s* distances is visualized in Figure 2a and pairwise *F_ST_* is visualized in Figure 2b.

Differences between some pairs of localities were consistent in all analyses with the Vr locality showing statistically significant pairwise differences with all analyzed localities. Statistically significant differences between localities were observed for pairs of the south (L-SP and L-T), all north and south/north (L-FG, L-DP, L-S, V-S, SP-S, and SP-DP) comparisons (Figure 1, Table S8). Overall, the north showed greater population differences than the southern regions, and there is no clear pattern of differentiation between the geographical regions.

The AMOVA performed across all 14 loci showed a low but significant value of genetic variance between localities (0.047), with 1.42% of the genetic variance being attributed to the variation among localities (Table 3). When localities were grouped according to their geographical region, the percentage of variation was higher within regions than among them. Additionally, when localities were grouped according to their region, the percentage of variation among localities within regions remained statistically significant, while differentiation between geographical regions could not be observed (Table S9).

**Figure 3:**
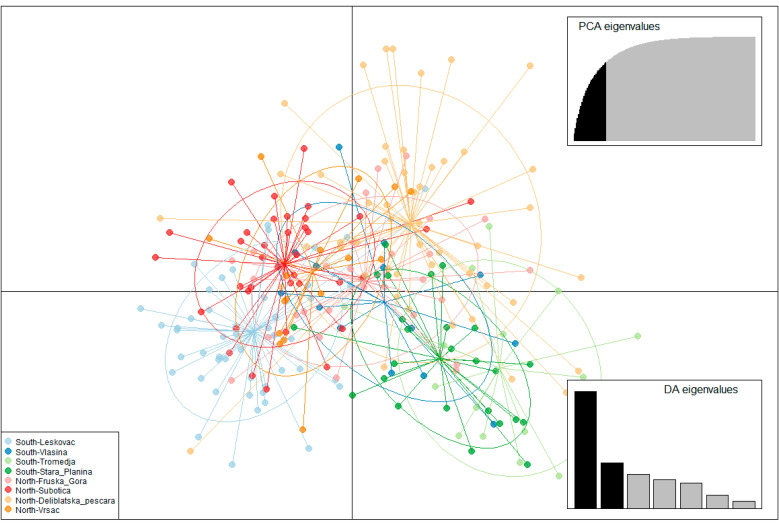
Discriminant analysis of principal components. The first and second linear discriminants are presented in the plot.

**Figure 4:**
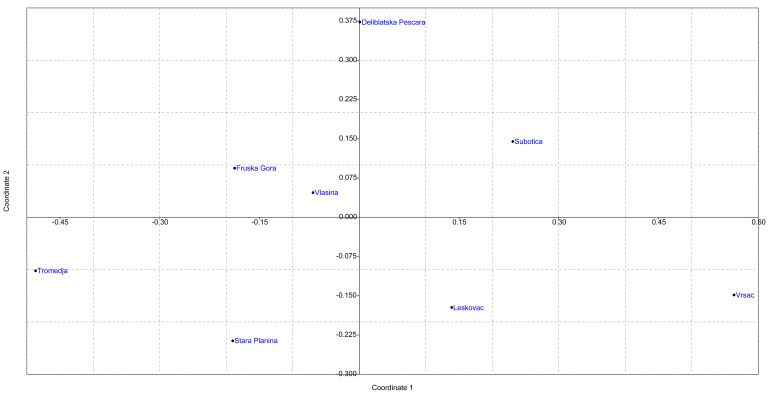
Non-metric multidimensional scaling plot of *F_ST_* distances between localities in Serbia. The goodness of fit is expressed with the stress value which is 0.1519 for this data set. Population pairwise *F_ST_* values are presented in Table S8.

The results of the analysis performed by the DAPC method are shown in Figure 3, Supplementary Figure S1, and the visualization by the MDS plot shows the positioning of populations in two dimensions (Figure 4). Although individuals from T, SP, L, and DP tend to cluster separately from others, DAPC analysis showed that individuals from geographically remote localities are grouped in cluster overlaps, indicating similarity between them. Moreover, assignment to the previous predesigned group was relatively low, with *p* ranging 0.2–0.3, indicating admixture. The MDS plot placed localities separately from each other, which is in correlation with AMOVA, suggesting the presence of distinct genetic variability in all analyzed localities. However, there is no clear grouping of localities according to their geographical region, which is also in concordance with AMOVA.

The STRUCTURE Harvester showed that *K* = 2 is the most likely scenario (Supplementary Figure S2). The same number of clusters was inferred by LnP (D) analysis (Supplementary Figure S3). Both clusters inferred by STRUCTURE are equally distributed in all sampled localities. Additionally, the number of clusters inferred with the DAPC method was 8, with mixed distribution across localities (Supplementary Figure S4).

#### 3.3.2. Population Structure Based on Nine Microsatellite Loci in All Sampled Localities

**Figure 5:**
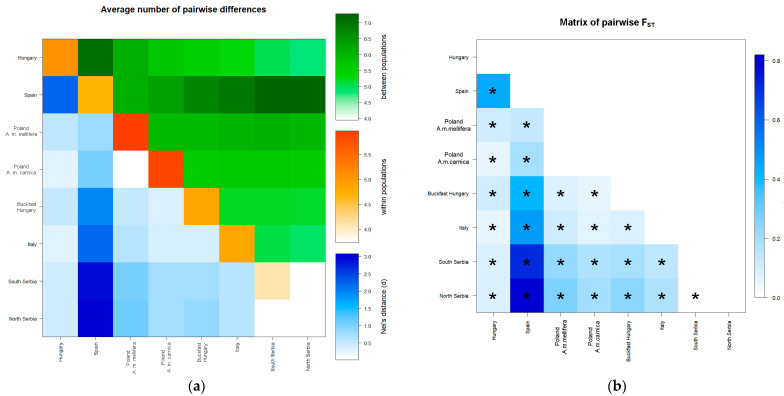
Matrixes of the average number of pairwise *Nei’s* (**a**) and *F_ST_* (**b**) distances based on the analysis of 9 microsatellite loci when all localities were grouped according to geographical region and subspecies. (**a**) The average number of pairwise differences between populations is presented above diagonally, the average number of pairwise differences within the population is presented diagonally, and *Nei’s* distances are presented below diagonally. (**b**) Statistically significant *F_ST_* values are marked with an asterisk (*).

The average number of pairwise differences between and within localities grouped according to their geographical region and subspecies together with *Nei’s* distances is visualized in Figure 5a, and pairwise *F_ST_* is visualized in Figure 5b. As expected, individuals from Spain were shown to be the most separated from others, but the separation between region and subspecies can also be observed since statistically significant *F_ST_* values were obtained (Table S10). The same conclusion can be inferred when all sampled localities were compared separately (Supplementary Figures S5 and S6, and Table S11). It is interesting to note that some differences between Serbian localities disappear but that there is a clear distinction between Serbian localities and other analyzed localities and subspecies. Moreover, very low but statistically significant *F_ST_* value was detected between southern and northern Serbia.

The AMOVA performed across nine loci showed a high and significant value of genetic variance among localities (0.28) with 10.79% of the genetic variance being attributed to the variation among the localities (Table 4). A negative value of differences among individuals within localities indicates that individuals in any given sampled population are mostly uniform and closely related to each other. Additionally, when AMOVA was performed with a different grouping of localities and subspecies, differences among geographical regions and subspecies remained significant, indicating regional differentiation that reflects subspecies and geographical distribution (Table S12).

**Figure 6:**
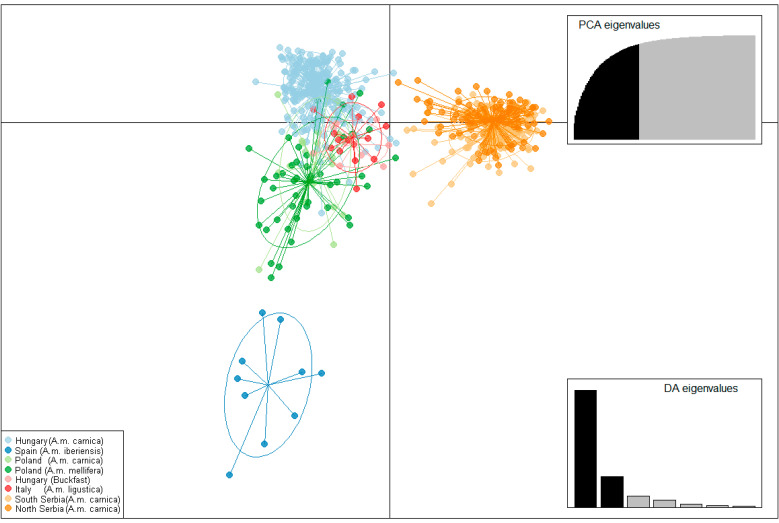
Discriminant analysis of principal components when all localities were grouped according to their geographical region and subspecies. The first and second linear discriminants are presented in the plot.

**Figure 7:**
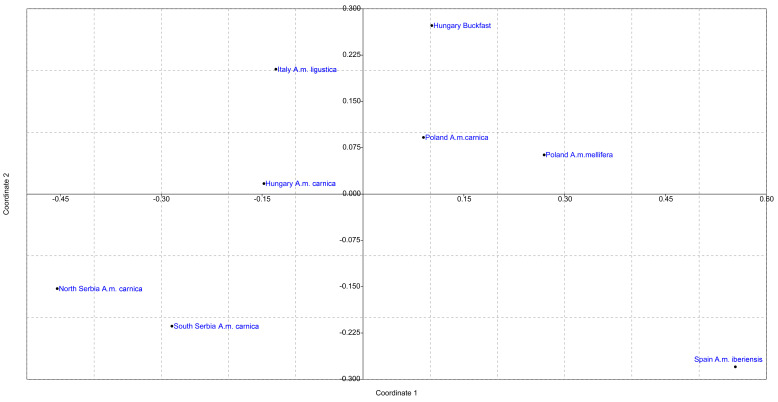
Non-metric multidimensional scaling plot of *F_ST_* distances between analyzed geographical regions and subspecies. The goodness of fit is expressed with the stress value which is 0.0426 for this data set. Population pairwise *F_ST_* values are presented in Table S10.

The results of AMOVA were further corroborated by DAPC analysis (Figure 6 and Supplementary Figure S7) and the positioning of the populations in two dimensions in the MDS plot (Figure 7). When localities were grouped according to geographical region and subspecies, clear differentiation could be observed. As expected, Spain’s population is the most separated from the others. Buckfast individuals from Hungary are closer to Italian individuals than Hungarian ones, and the Hungarian population is relatively homogeneous as previously reported. Serbian localities were in a cluster overlap and separated from other analyzed populations. The alternative grouping of localities and subspecies does not change the relative relations among the analyzed localities; localities from the same geographical region and subspecies were always clustered together and separated from others (Supplementary Figures S5–S7).

**Figure 8:**
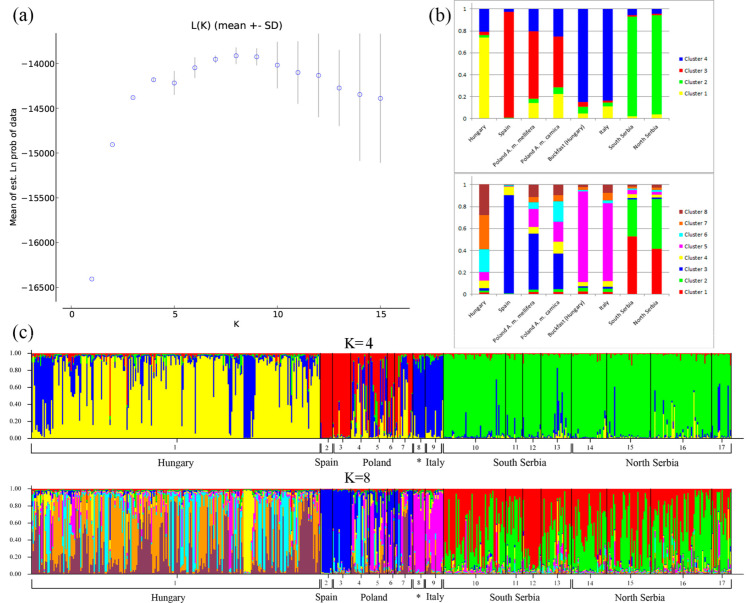
(**a**) L(K) mean for the assumed number of genetic clusters. (**b**) Proportions of inferred STRUCTURE clusters (*K* = 4 and *K* = 8). (**c**) Proportions of the inferred STRUCTURE clusters (*K* = 4 and *K* = 8) from the individuals. 1—Hungary, 2—Spain, 3–7—Poland (3—August forest, 4—Krakow, 5—Bialowieza, 6—Wrocław, and 7—Siedlice), * 8—Buckfast lineage from Hungary, 9—Italy, 10–13—Southern Serbia (10—Leskovac, 11—Vlasina, 12—Tromeđa, and 13—Stara Planina), and 14–17—Northern Serbia (14—Fruška Gora, 15—Subotica, 16—Deliblatska peščara, and 17—Vršac).

The STRUCTURE Harvester showed that *K* = 8 is the most likely scenario (Figure 8), since the LnP (D) showed that *K* = 8 best fits the data even though Δ K suggested *K* = 2 has the highest probability (Figure S8). In our data, *K* = 8 gives the most plausible distribution of inferred genetic clusters, which were specifically distributed among the individuals in the populations originating from different geographical regions or subspecies. Furthermore, the number of clusters inferred with the DAPC method was four, with specific distribution of clusters across sampled geographical regions (Supplementary Figure S9).

Based on all analyses it can be concluded that strong geographical differentiation exists between analyzed geographical regions and their corresponding subspecies.

## 4. Discussion

The modernization of beekeeping practices and rapidly growing numbers of beehives in Serbia have invoked the need to re-study previously described genetic variability in the Serbian honey bee population [23,24,26,44,45,53]. Most of the previous genetic studies on the Serbian honey bee, even the most recently published ones, are based on samples from the first decade of the 21st century. Since then, significant changes in beekeeping practices together with stricter implementation of Serbian legislation and an increased number of beehives have led to changes in genetic variability in Serbian honey bee populations, as suggested in [46,61]. Therefore, we examined 14 microsatellite loci in Serbian worker bees from eight different localities to further shed light on the current status of the genetic diversity of Serbian honey bees. Furthermore, we compared nine microsatellite loci in our sample with previously published samples from Hungary, Poland, Spain, and Italy [58] to infer broader genetic relations between different *A. mellifera* populations and subspecies. Our results suggest that the Serbian honey bee population is relatively homogenous with preserved local differences and separated from the other populations analyzed.

The parameters of genetic diversity in Serbian localities are relatively high but lower than in other analyzed localities. Moreover, reference localities showed significant heterozygosity excess, while in Serbian localities Ho was in line with He, and for some loci, heterozygote deficiency may have been observed. These results, together with the G–W index, indicate that the Serbian honey bee population did not experience recent bottleneck events. For some loci, departures from Hardy–Weinberg and linkage disequilibrium were observed, which may be an indication of recent gene flow from other subspecies or populations [73].

Although significant *F_ST_* values were obtained between some pairs of localities, there is no clear pattern that indicates a south/north geographical distribution of microsatellite loci in the Serbian honey bee population. The results of AMOVA analysis suggest that grouping according to region may indicate some geographical distribution since the percentage of variation among localities within groups slightly decreases, but the value is not statistically significant (0.32, *p* = 0.119). Together with the equal distribution of two clusters inferred by STRUCTURE analysis in all localities and cluster overlapping inferred by DAPC analysis, the presented results indicate population admixture and a relatively homogenous population. However, local differences are still preserved since significant *F_ST_* values can be observed between pairs of localities, and some differentiation may be observed according to the position of population in DAPC and MDS landscapes.

Our results could not confirm the presence of *A. m. macedonica* and north/south differences between individuals from different parts of the country previously reported for the Serbian honey bee population [23,26,53] but are in concordance with recently published work on the genetic diversity of the mtDNA tRNA^leu^-cox2 for the same sample [46]. Both uniparental and biparental markers showed that although local specific genetic variants and weak regional differences can be observed, previously reported regional differences indicative of subspecies distribution could not be confirmed. The formation of an admixture population may be one of the reasons behind the presented results. Extensive hybridization between *A. m. carnica* and *A. m. macedonica* subspecies in the central part of Serbian territory was previously described [23,26], and it is possible that the hybridization zone expanded reflecting recent changes in beekeeping practices as was shown by mtDNA data. The absence of north/south regional differentiation may also be partially attributed to the intensification of migratory beekeeping, since apiaries from the south are transported to the north during the flowering season of agricultural plants. As there is no human control of mating between individuals from different apiaries, when migratory apiaries return to the region where the stationary apiaries sampled in this study are located, admixture may be propelled. The Serbian leading beekeeper organization strongly encourages strict implementation of Serbian legislation that only *A. m. carnica* subspecies can be present in apiaries which, with a growing number of *A. m. carnica* queen manufacturers, may also contribute to the observed loss of *A. m. macedonica* and the admixture of the Serbian honey bee population.

Population structure analysis showed that each geographical region and each corresponding subspecies are separated. *A. m. iberica* from Spain is represented by its own cluster in STRUCTURE analysis as well as clearly separated in MDS and DAPC plots. The Italian *A. m. ligustica* is represented by its own structure cluster which is present in the Hungarian Buckfast sample as expected. The same result was obtained from DAPC and MDS analysis. The Polish populations of *A. m. carnica* and *A. m. mellifera* were located close to each other but still separated. The Hungarian population is well separated from the other populations studied, and although relatively homogeneous, some *A. m. ligustica* introgression may be observed, as previously reported by [58]. Serbian populations are well separated from others with significant overlap between individuals from the south and north, although very low but significant *F_ST_* value can be observed. Structure analysis showed weak but still detectable introgression of *A. m. ligustica* alleles, which is in concordance with our field data that some illegal importation of Italian bees occurred in the past, since this subspecies has been one of beekeepers’ favorites. Two distinct clusters can be observed in the Serbian honey bee population, and they are almost equally distributed among localities, suggesting population admixture.

Our results suggest that, as already shown in many studies [74], geographical distance together with environmental factors maintain the specific genetic diversity of *A. mellifera* subspecies within any given geographical region. However, this genetic diversity is under constant anthropogenic influence due to the modernization of beekeeping practices, such as migratory beekeeping, importation of foreign queens, and even legal practices [4,33,35,37]. Serbia is the natural area of contact between warmer climates preferring *A. m. macedonica* and colder ones preferring *A. m. carnica* and, although relief and ecological differences exist between these two parts of the country, climate conditions are favorable for both subspecies, and the main reason between their distinct distribution may lie in isolation by distance. The distances between the southern and northern parts of Serbia may be too great for bees, but for beekeepers they are rather small and they readily travel 500 km in the flowering season for different plants. Together with a vast increase in the number of beehives and beekeepers in the past decade [54] and legislation that specifically allows breeding of a single subspecies, it is not surprising that the previous composition of the diversity of honey bees in Serbia has changed. However, specific local genetic variability may still be retained since differences between analyzed localities can be observed.

Unfortunately, socioeconomic interests and beekeeper preferences for more productive and gentler individuals have often taken precedence over the conservation of locally native subspecies [27,37,39], leading to the predominance of admixture populations in human-dominated areas [4]. Our results suggest that this scenario happened in Serbian honey bee populations and that for the above-mentioned reasons Serbia now harbors a distinct hybrid honey bee population. Further analysis that will include honey bee populations from eastern and western parts of Serbia are needed in order to better understand the pattern of genetic variability of managed honey bees in Serbia, so that the best managing strategies, with the goal of preserving the existing genetic diversity, can be implemented.

## Figures and Tables

**Table 1:** Standard diversity parameters for sampled localities in Serbia for 14 microsatellite loci.

Locality	N	Na	Agd	Ho	He	Ar	RMP	MPD	G-W	Ar8	Apr
Leskovac (L)	51	9	13.571	0.5984	0.5617	0.5958	0.00980	7.7786	0.6638	3.44	0.37
Vlasina (V)	14	5.7140	8.429	0.5584	0.5192	0.5888	0.03570	6.7011	0.6350	3.41	0.33
Tromedja (T)	15	5.1430	7.929	0.5184	0.4698	0.5397	0.03330	6.2207	0.6605	3.05	0.21
Stara Planina (SP)	25	6.7860	8.643	0.5559	0.5291	0.5785	0.02000	5.5592	0.6890	3.38	0.35
Fruska Gora (FG)	29	7.0710	10.714	0.5063	0.4662	0.5444	0.01780	5.5693	0.6163	3.2	0.26
Subotica	37	7.2140	10	0.5510	0.5054	0.5552	0.01350	7.7138	0.6667	3.19	0.22
Deliblatska Pescara (DP)	50	8.2140	13.071	0.5121	0.5098	0.5734	0.01020	4.0968	0.6096	3.3	0.34
Vrsac (Vr)	16	5.7860	8.500	0.5783	0.4311	0.5674	0.03120	6.9395	0.6516	3.28	0.35

N—number of genotyped individuals, Na—the average number of alleles, Ar—allelic size range, Agd—average gene diversity over loci, Ho—observed heterozygosity, He—expected heterozygosity, RMP—random match probability, MPD—mean number of pairwise differences, G–W—Garza–Williamson index, Ar8—number of alleles based on a minimal sample size of 8 diploid individuals, and Apr—number of private alleles based on a sample of 8 diploid individuals. Part of the results are published in Proceedings of the The 1st International Electronic Conference on Entomology session Apiculture and Pollinators, 1–15 July 2021, MDPI: Basel, Switzerland, doi: 10.3390/IECE-10720.

**Table 2:** Standard diversity parameters for all analyzed localities for 9 microsatellite loci.

Locality	N	Na	Agd	Ho	He	Ar	RMP	MPD	G-W	Ar10	Apr
Hungary (*A. m. carnica*)	237	14	0.63694	0.89613	0.65753	35	0.0025	5.7325	0.5158	3.85	0.27
Spain (*A. m. iberiensis*)	10	5.3	0.73895	0.81687	0.64477	9.375	0.0500	3.6947	0.62704	4.18	1.22
Poland (*A. m. mellifera*)	45	9.3	0.75006	0.86408	0.75869	12.44	0.0111	6.7506	0.70839	4.62	0.36
Poland Aug forest	15	6.4	0.71239	0.88148	0.71239	7.667	0.0333	6.4115	0.74374	4.23	0.23
Poland Bialowieza	15	7.4	0.74738	0.82222	0.74738	11.667	0.0333	6.7264	0.63242	4.66	0.21
Poland Siedlice	15	6.1	0.75603	0.88889	0.75648	7.556	0.0333	6.0483	0.75483	4.35	0.06
Poland (*A. m. carnica*)	21	7.6	0.72887	0.90476	0.74671	9.000	0.0249	6.5598	0.78886	4.49	0.37
Poland Krakow	15	6.9	0.74253	0.9037	0.73498	8.667	0.0333	5.9402	0.76265	4.47	0.19
Poland Wroclaw	6	3.8	0.70076	0.90741	0.70932	5.222	0.0972	5.6061	0.65743	3.63	0.03
Buckfast (Hungary)	10	4.3	0.63158	0.84321	0.64419	7.556	0.05	5.0526	0.55482	3.57	0.13
Italy (*A. m. ligustica*)	15	5.3	0.60977	0.91111	0.6349	6.778	0.0333	4.8782	0.69998	3.58	0.08
Southern Serbia (*A. m. carnica*)	105	10.2	0.51083	0.46459	0.52209	14.67	0.0051	4.0866	0.69461	3.47	0.48
Serbia Leskovac	51	8.6	0.54021	0.50215	0.53994	12.444	0.0106	4.3217	0.68713	3.52	0.21
Serbia Vlasina	14	5.3	0.50970	0.44445	0.50970	8.222	0.0357	4.5873	0.64462	3.38	0.13
Serbia Tromedja	15	4.8	0.42644	0.41235	0.44805	7.889	0.0333	3.4115	0.63851	2.98	0.08
Serbia Stara Planina	25	6.7	0.46898	0.42468	0.51138	9.222	0.0208	2.8139	0.62132	3.44	0.20
Northern Serbia (*A. m. carnica*)	131	10.4	0.46634	0.40063	0.47838	13.333	0.0044	3.7307	0.72894	3.23	0.36
Serbia Fruska Gora	29	6	0.38355	0.38641	0.438	9.111	0.0196	2.6848	0.5726	3.02	0.08
Serbia Subotica	36	6.7	0.47344	0.44136	0.47344	9.556	0.0143	4.261	0.6193	3.11	0.06
Serbia Deliblatska Pescara	50	7.9	0.3918	0.40292	0.49063	12.889	0.0104	1.959	0.6065	3.28	0.13
Serbia Vrsac	16	5.2	0.49568	0.33029	0.49716	8.667	0.0312	3.4698	0.59755	3.23	0.26

N—number of genotyped individuals, Na—the average number of alleles, Ar—allelic size range, Agd—average gene diversity over loci, Ho—observed heterozygosity, He—expected heterozygosity, MPD—mean number of pairwise differences, RMP—random match probability, G–W—Garza–Williamson index, Ar10—number of alleles based on a minimal sample size of 10 diploid individuals, and Apr—number of private alleles based on a sample of 10 diploid individuals.

**Table 3:** AMOVA results when all localities in Serbia were analyzed without grouping.

Source of Variation	d.f.	SS	Variance Components	Percentage of Variation
Among localities	7	43.424	0.047	1.42 (***p* = 0.005**)
Among individuals within localities	229	797.685	0.184	5.49 (***p* = 0.0001**)
Within individuals	237	738.685	3.116	93.1 (***p* = 0.000**)
Total	473	1579.61	3.347	

d.f.—degrees of freedom, SS—the sum of squares, and *p*-statistical significance (statistically significant values are in bold).

**Table 4:** AMOVA results when all localities were analyzed without grouping.

Source of Variation	d.f.	SS	Variance Components	Percentage of Variation
Among localities	16	286.781	0.2801	**10.79 (*p* = 0.00)**
Among individuals within localities	557	1034.257	−0.4601	−17.72
Within individuals	574	1594.000	2.7770	106.93
Total	1147	2915.037	2.5970	

d.f.—degrees of freedom, SS—the sum of squares, and *p*-statistical significance (statistically significant values are in bold).

## Data Availability

Data are available on request from the corresponding author.

## Supplementary Materials

The following supporting information can be downloaded at: https://www.mdpi.com/article/10.3390/insects13020180/s1, Figure S1: Discriminant analysis of principal components. The first three linear discriminants are presented in the plot; Figure S2: (a) Delta *K* values for the assumed number of genetic clusters. (b) Proportions of inferred STRUCTURE clusters (*K* = 2). (c) Proportions of the inferred STRUCTURE clusters (*K* = 2) from the individual worker bees; Figure S3: Ln values of probability for the assumed number of genetic clusters; Figure S4: Distribution of clusters according to the DAPC method and inffered number of 8 clusters; Figure S5: Non-metric multidimensional scaling plot of *F_ST_* distances between 8 localities in Serbia (*A. m. carnica*) and other analysed *A. mellifera* populations from Hungary (*A. m. carnica* and Buckfast), Poland (Krakow and Wroclaw–*A. m. carnica*; August forest, Bialowieza and Siedlice–*A. m. mellifera*) Spain (*A. m. iberica*) and Italy (*A. m. ligustica*); Figure S6: Non-metric multidimensional scaling plot of *F_ST_* distances between 8 localities in Serbia (*A. m. carnica*) and other analysed *A. mellifera* populations from Hungary (*A. m. carnica* and Buckfast), Poland (Krakow and Wroclaw–*A. m. carnica*; August forest, Bialowieza and Siedlice–*A. m. mellifera*) Spain (*A. m. iberica*) and Italy (*A. m. ligustica*); Figure S7: Discriminant analysis of principal components. The first three linear discriminants are presented in the plot; Figure S8: Delta *K* values for the assumed number of genetic clusters; Figure S9: Distribution of clusters according to the DAPC method and inffered number of 4 clusters; Table S1: List of loci used in genetic analyses, primers used for their amplification, fluorescent dyes used for tagging the forward primers, annealing temperature (Tm) used in each reaction for the amplification of specific microsatellite loci with the combination of loci amplified together in multiplex reactions I, II, III and IV; Table S2: Parameters of genetic diversity calculated per locus per populations including Garza Williamson index, Hardy Weinberg equilibrium (1,001,000 steps done)–Serbian localities; Table S3: Population specific Fis indices for 10,100 permutations (Serbian localities); Table S4: Tables of significant linkage disequilibrium (Serbian localities); Table S5: Parameters of genetic diversity calculated per locus per populations including Garza Williamson index, Hardy Weinberg equilibrium (1,001,000 steps done) (all populations); Table S6: Population specific Fis indices for 10,100 permutations (all populations); Table S7: Tables of significant linkage disequilibrium (all populations); Table S8. Pairwise population Fst (below diagonal) and Fst *p* values (above diagonal) between the populations based on the variability of 14 microsatellite loci found in 8 different localities of *Apis mellifera carnica* in Serbia; Table S9: Outcomes of AMOVA analysis based on the variability of 14 microsatelite loci when population sample from Serbia was grouped according to the geographical region: North vs South; Table S10: Pairwise population Fst (below diagonal) and Fst *p* values (above diagonal) between the populations based on the variability of 9 microsatellite loci when Serbian localities were grouped according to their geographical region and other *A. mellifera* populations from: Hungary (*A. m. carnica* and Buckfast), Poland (*A. m. carnica* and *A. m. mellifera*), Italy (*A. m. ligustica*) and Spain (*A. m. iberica*); Table S11: Pairwise population Fst (below diagonal) and Fst *p* values (above diagonal) between the populations based on the variability of 9 microsatellite loci found in 8 different localities of *Apis mellifera carnica* from Serbia and other *A. mellifera* populations from: Hungary (*A. m. carnica* and Buckfast), Poland (*A. m. carnica* and *A. m. mellifera*), Italy (*A. m. ligustica*) and Spain (*A. m. iberica*); Table S12: Outcomes of AMOVA analysis based on the variability of 9 microsatelite loci when population sample from Serbia was compared with other analysed populations.
