# Supplementary material for: Further Evidence of Population Admixture in the Serbian Honey Bee Population
Source: Insects. 2022 Feb 9;13(2):180. doi: 10.3390/insects13020180 (PMC8879341; doi:10.3390/insects13020180)
Supplement: Supplementary file 1 [file insects-13-00180-s001.zip › Supplementary figure S1-S9.pdf]

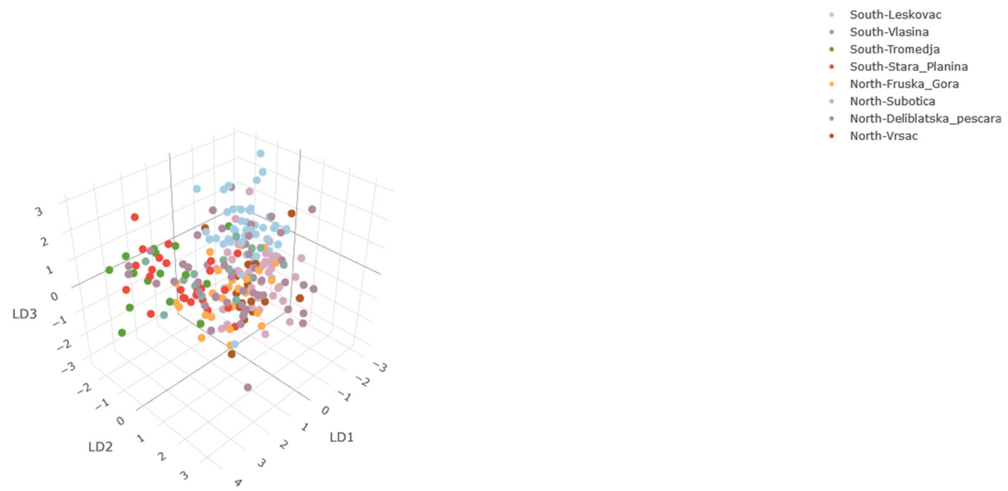


**Figure S1.** Discriminant analysis of principal components. The first three linear discriminants are presented in the plot.

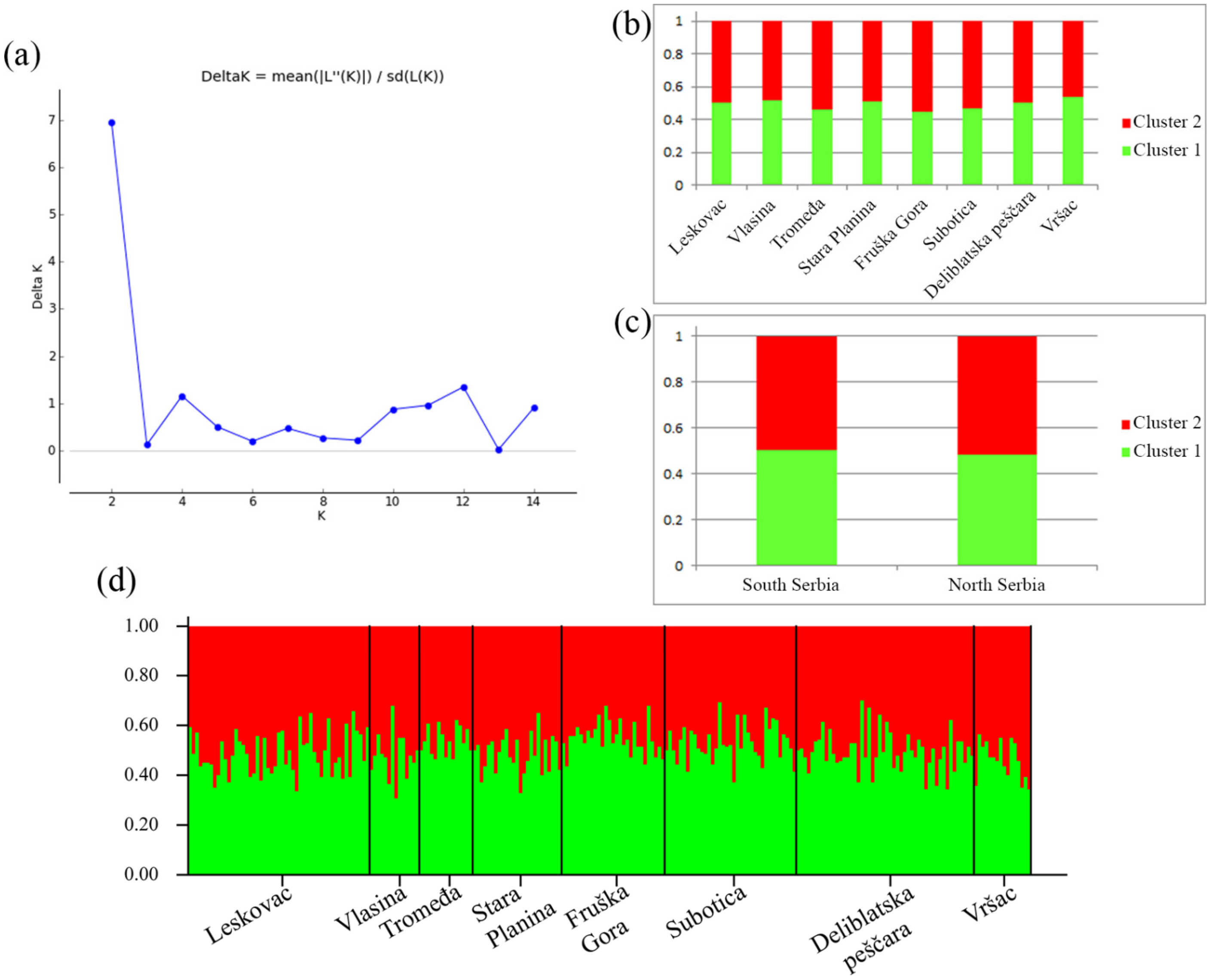


**Figure S2.** (a) Delta K values for the assumed number of genetic clusters. (b) Proportions of inferred STRUCTURE clusters (K = 2). (c) Proportions of the inferred STRUCTURE clusters (K = 2) from the individual worker bees. (d) Proportions of the inferred STRUCTURE clusters ($K$ = 2) from the individuals.

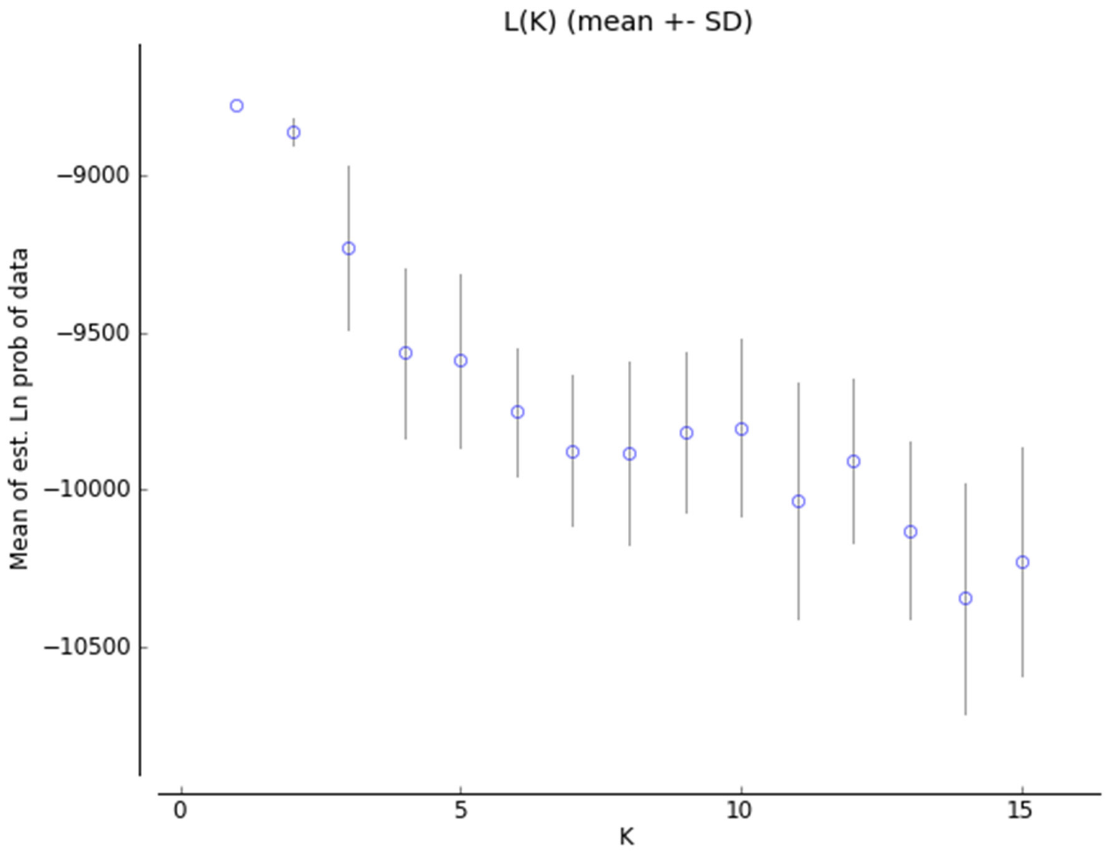


**Figure S3.** Ln values of probability for the assumed number of genetic clusters.

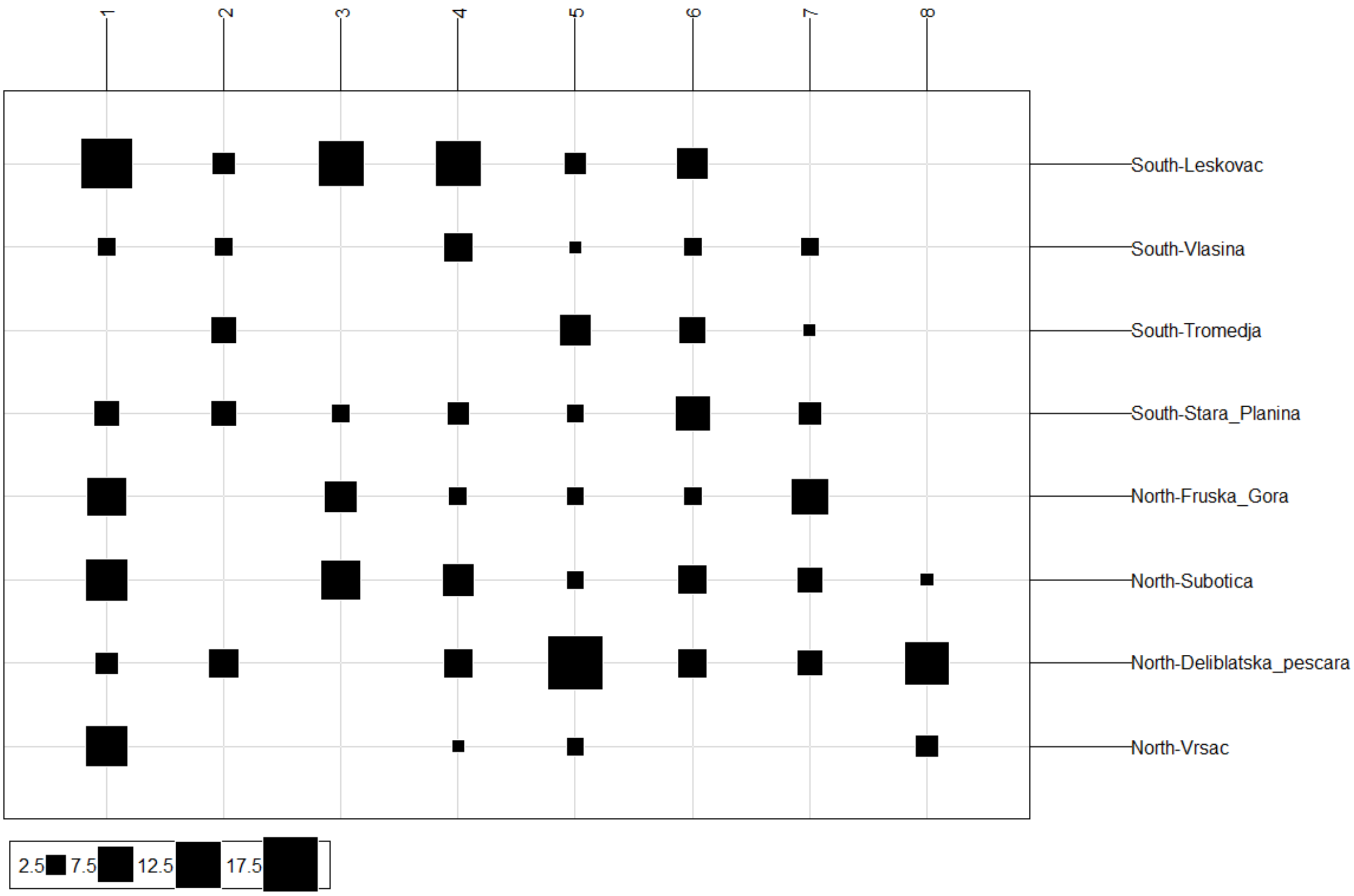


**Figure S4.** Distribution of clusters according to the DAPC method and inffered number of 8 clusters.

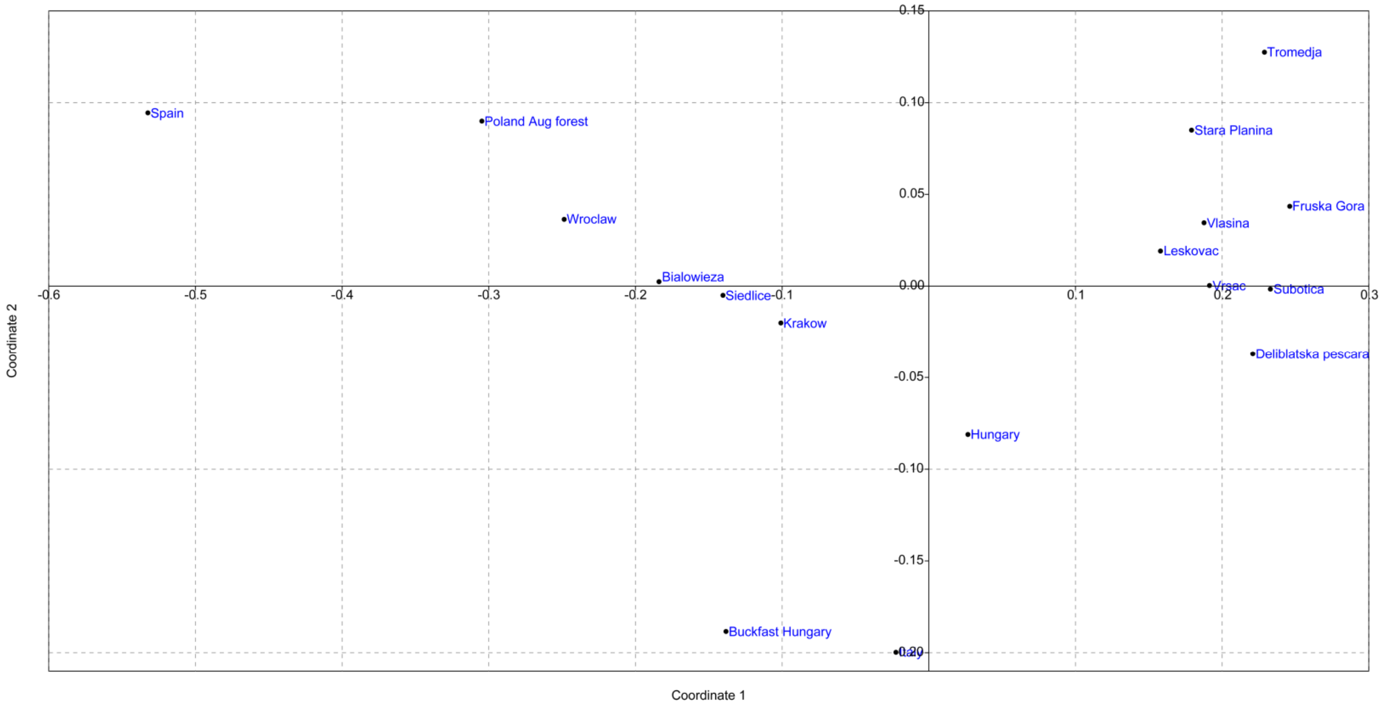


**Figure S5.** Non-metric multidimensional scaling plot of $F_{ST}$ distances between 8 localities in Serbia (*A. m. carnica*) and other analysed *A. mellifera* populations from Hungary (*A. m. carnica* and Buckfast), Poland (Krakow and Wroclaw - *A. m. carnica*; August forest, Bialowieza and Siedlice – *A. m. mellifera*) Spain (*A. m. iberica*) and Italy (*A. m. ligustica*) . The goodness of fit is expressed with the stress value which is 0.07772 for this data set. Population pairwise $F_{ST}$ values are presented in Table S11a.

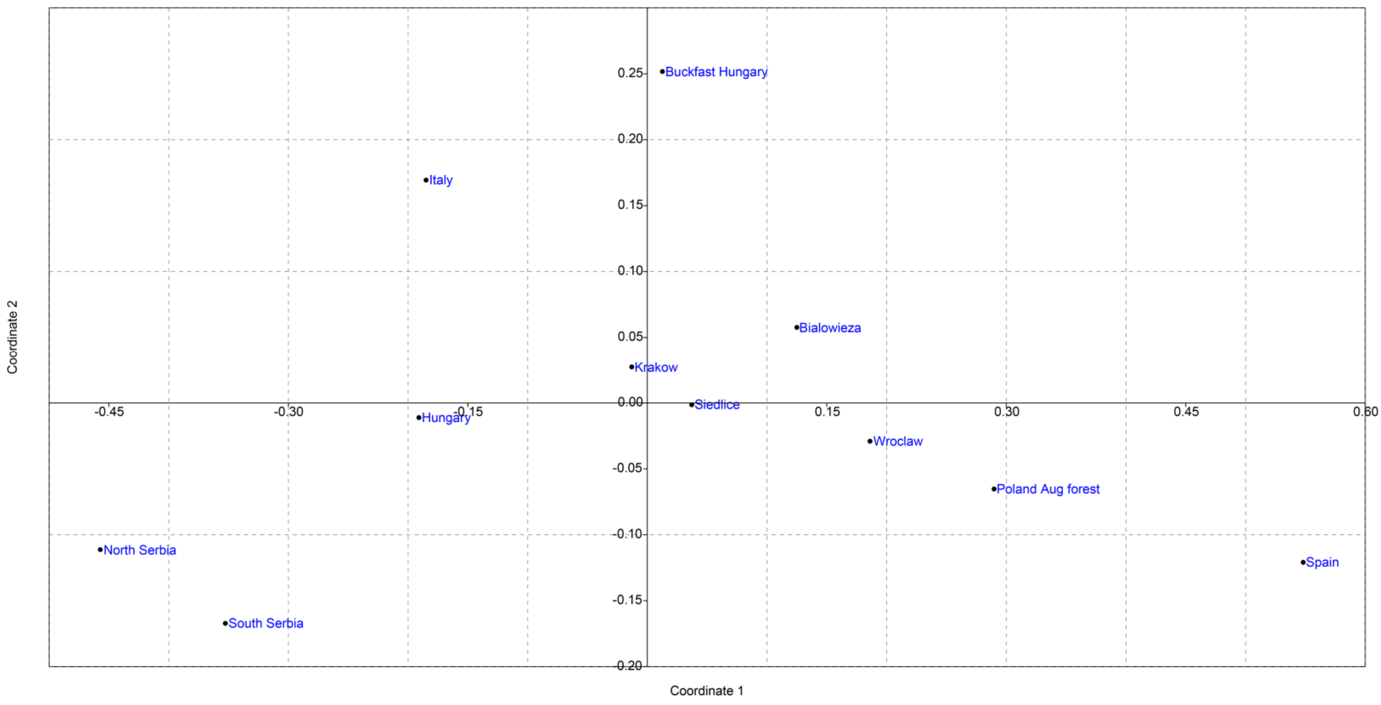


**Figure S6.** Non-metric multidimensional scaling plot of $F_{ST}$ distances between 8 localities in Serbia (*A. m. carnica*) and other analysed *A. mellifera* populations from Hungary (*A. m. carnica* and Buckfast), Poland (Krakow and Wroclaw - *A. m. carnica*; August forest, Bialowieza and Siedlice – *A. m. mellifera*) Spain (*A. m. iberica*) and Italy (*A. m. ligustica*) . The goodness of fit is expressed with the stress value which is 0.08112 for this data set. Population pairwise $F_{ST}$ values are presented in Table S11b.

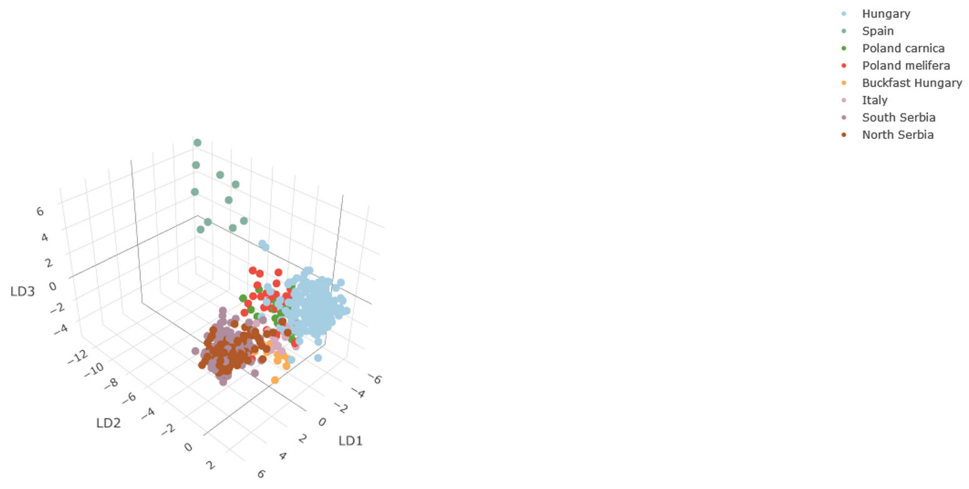


**Figure S7.** Discriminant analysis of principal components. The first three linear discriminants are presented in the plot.

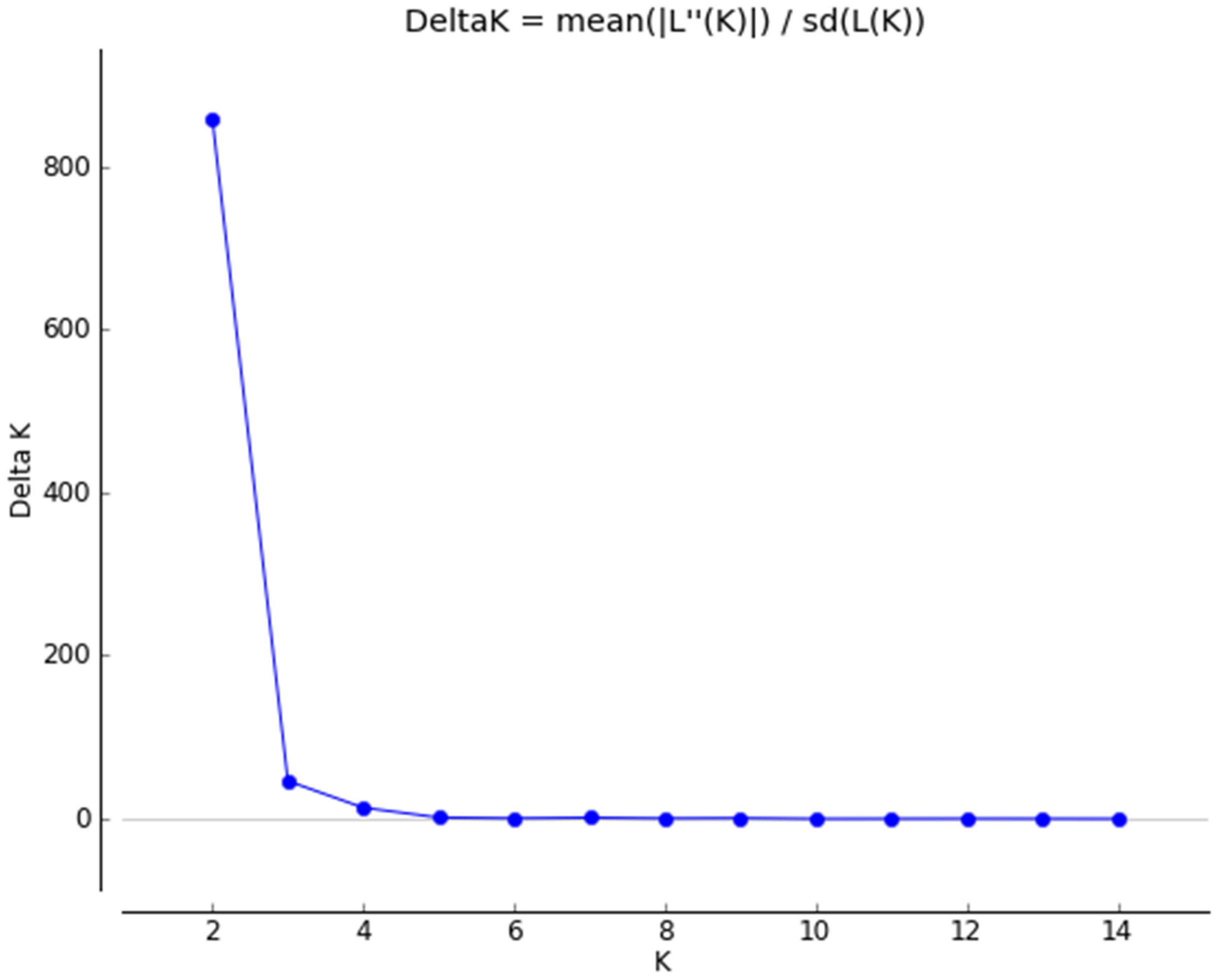


**Figure S8.** Delta *K* values for the assumed number of genetic clusters.

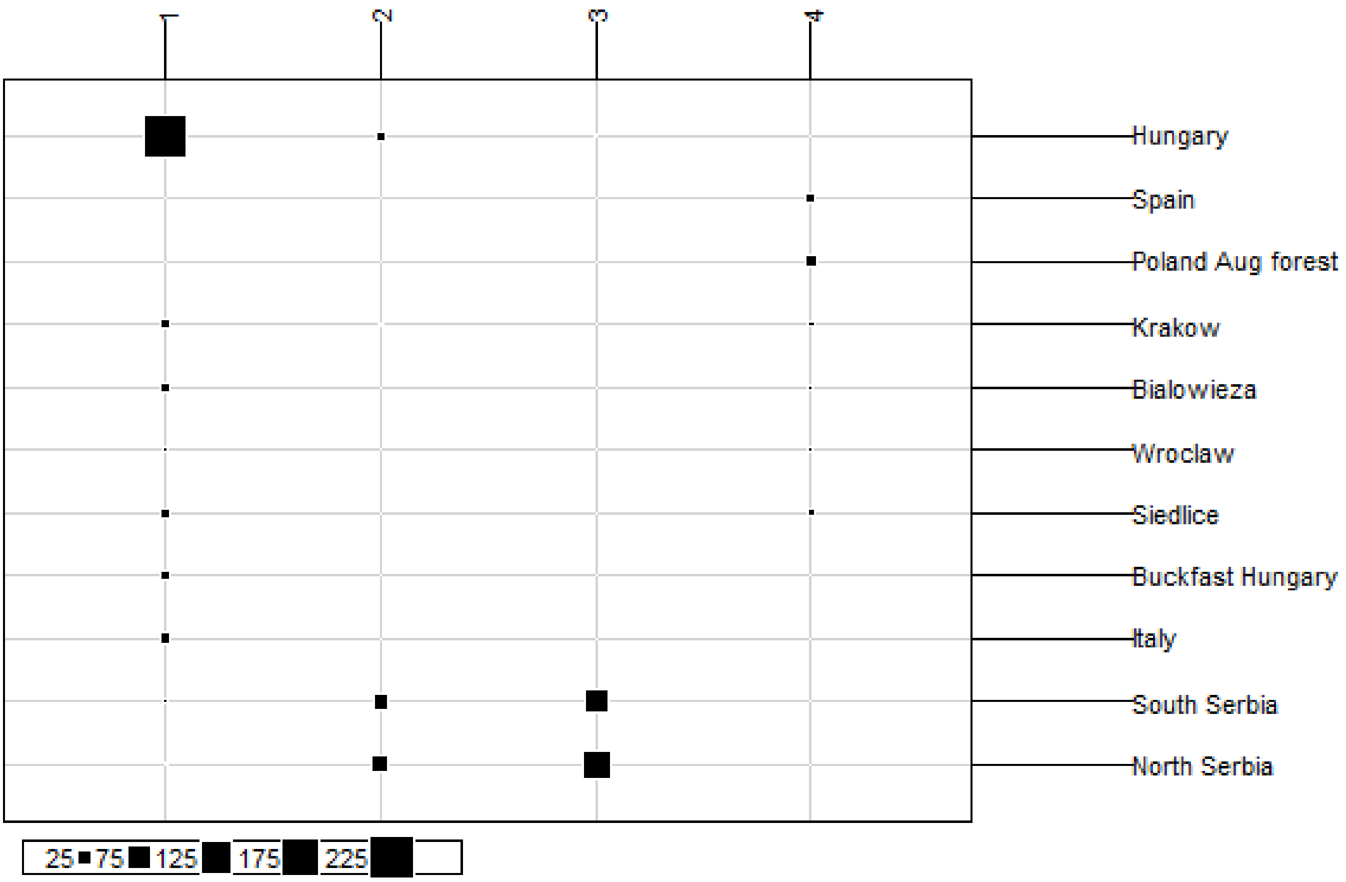


**Figure S9.** Distribution of clusters according to the DAPC method and inffered number of 4 clusters.
